# Effects of Graphene Oxide Nanoparticles on the Immune System Biomarkers Produced by RAW 264.7 and Human Whole Blood Cell Cultures

**DOI:** 10.3390/nano8020125

**Published:** 2018-02-24

**Authors:** Kim Lategan, Hend Alghadi, Mohamed Bayati, Maria Fidalgo de Cortalezzi, Edmund Pool

**Affiliations:** 1 Department of Medical Bioscience, University of the Western Cape, Cape Town 7535, South Africa; 2917132@myuwc.ac.za (K.L.); hendalghadi@gmail.com (H.A.); 2 Department of Civil and Environmental Engineering, University of Missouri, Columbia, MO 65211, USA; mbb229@mail.missouri.edu (M.B.); fidalgom@missouri.edu (M.F.d.C.)

**Keywords:** graphene oxide nanoparticles, cytotoxicity, macrophage activation, humoral immune response

## Abstract

Graphene oxide nanoparticles (GONPs) have attracted a lot of attention due to their many applications. These applications include batteries, super capacitors, drug delivery and biosensing. However, few studies have investigated the effects of these nanoparticles on the immune system. In this study, the in vitro effects of GONPs on the immune system was evaluated by exposing murine macrophages, RAW 264.7 cells and human whole blood cell cultures (to GONPs. The effects of GONPs on RAW cells were monitored under basal conditions. The whole blood cell cultures were exposed to GONPs in the presence or absence of the mitogens lipopolysaccharide (LPS) and phytohaemmagglutinin (PHA). A number of parameters were monitored for both RAW and whole blood cell cultures, these included cytotoxicity, inflammatory biomarkers, cytokines of the acquired immune system and a proteome profile analysis. The GONPs were cytotoxic to both RAW and whole blood cell cultures at 500 μg/mL. In the absence of LPS, GONPs elicited an inflammatory response from the murine macrophage, RAW and whole blood cell cultures at 15.6 and 5 μg/mL respectively. This activation was further corroborated by proteome profile analysis of both experimental cultures. GONPs inhibited LPS induced interleukin 6 (IL-6) synthesis and PHA induced interferon gamma (IFNγ) synthesis by whole blood cell cultures in a dose dependent manner. In the absence of mitogens, GONPs stimulated IL-10 synthesis by whole blood cell cultures. The current study shows that GONPs modulate immune system biomarkers and that these may pose a health risk to individuals exposed to this type of nanoparticle.

## 1. Introduction

Graphene (G) is a two-dimensional (2-D) carbon material, with a hexagonal structure (honeycomb lattice) consisting of sp^2^ hybridized atoms, which has been isolated from its three-dimensional parent material, graphite [1,2,3]. Graphene oxide (GO) is a single carbon layer graphene derivative, containing oxygen-bearing functional groups such as carboxyl and hydroxyl groups [2,4,5]. These groups are acquired when graphene undergoes oxidization [2]. These oxidized graphene nanoparticles possess unique characteristics such as electronic and thermal conductivity, superior mechanical strength and optical properties [6]. Due to these exceptional physiochemical properties of graphene oxide nanoparticles (GONPs), it has a broad range of applications in a number of fields. These fields include electrochemistry, biomedicine, biosensing, drug delivery, high energy capacity batteries and super capacitors to name a few. However, these properties do not necessarily ensure that GONPs are good drug delivery carriers [2,5].

Due to the applications of GONPs it is inevitable that these nanoparticles will be released into the environment and may directly or indirectly affect human health. Therefore, it is imperative that the effects of GO be investigated. As nanoparticles, due to their properties may be easily transported, which alters bioavailability and affects cellular toxicity [7]. GO was only discovered in 2004 and since its discovery, few studies have reported on its effects on the immune system [2]. Graphene oxide induced oxidative stress and immunotoxicity in vivo in zebrafish [1]. Upon in vitroexposure to GONPs, decrease in cell viability, DNA damage, increased reactive oxygen species (ROS) production and induction of inflammatory factors were recorded [4,8,9]. The cytokines selected in this in vitro study (i.e., IL-6, IL-10 and IFNγ) are indicative of the inflammatory, humoral and cell mediated immune systems respectively. They each play different but inter-connective roles. IL-6 has the ability to stimulate T cell proliferation and their differentiation into cytotoxic T cells, stimulating antibody production and the induction of acute phase proteins [10,11]. Whereas, IL-10 has the unique ability to inhibit the expression and production of many lipopolysaccharide (LPS)-inducible genes, mainly pro-inflammatory cytokines, by macrophages [12,13]. And IFNγ is produced mainly by activated CD4^+^ or CD8^+^ T cells and natural killer (NK) cells and is recognized as the principal mediator of innate and adaptive immunity [14,15]. Therefore, validating their selection as GONPs could potentially result in immunotoxicity. Studies also showed that GONPs exhibited antibacterial effects against both gram negative and gram positive bacteria [16].

The aim of current study was to investigate the in vitro effects of GONPs on the immune system by exposing the murine macrophage cell line, RAW 264.7 and human whole blood cell cultures to GONPs. A number of immune system biomarkers were monitored such as cytotoxicity, inflammatory biomarkers, cytokines of the acquired immune system and a proteome profile analysis of cytokines and chemokines expressed upon GONPs exposure.

## 2. Results

### 2.1. The Effects of GONPs on RAW 264.7 Cells

#### 2.1.1. The Effects of GONPs on Viability of RAW 264.7 Cells

**Figure 1:**
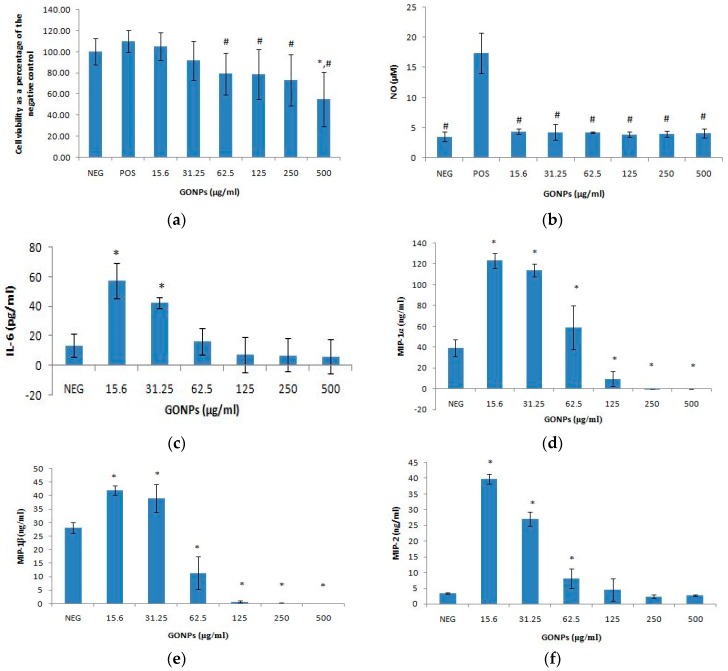
Murine macrophage, RAW 264.7 cells after treatment with GONPs. Parameters assessed (**a**) cell viability; (**b**) nitric oxide production; (**c**) Interleukin 6 (IL-6) production; (**d**) Macrophage inflammatory protein 1 alpha (MIP-1α) levels. Positive control not represented (803.85 ± 353.70 ng/mL MIP-1α); (**e**) MIP-1β levels. Positive control not represented (1127.19 ± 468.69 ng/mL MIP-1β); and (**f**) MIP-2 levels. Positive control not represented (307.39 ± 171.86 ng/mL MIP-2). Data represents mean ± SD with *n* = 9. Bars marked with symbols indicate significant difference to control (*p* < 0.01). Significance demarcated by: *—significantly different compared to negative control (*p* < 0.001), ^#^—significantly different compared to positive control (*p* ≤ 0.005).

GONPs at concentrations ≤ 31.25 μg/mL did not have an effect on cell viability (Figure 1a). However, GONPs concentrations ≥ 62.5 μg/mL significantly (*p* < 0.001) reduced cell viability. At the highest concentration of GONP screened (500 μg/mL), the viability of cells was less than 60% of the control cell viability (cells that did not receive GONP).

#### 2.1.2. The Effects of GONPs on the Inflammatory System Biomarker Nitric Oxide (NO) Using RAW 264.7 Cells

GONPs had no effect on the inflammatory response from the cells in an unstimulated environment across all the concentrations monitored in this study (Figure 1b). A LPS stimulated control without any nanoparticle is included in the results to verify that the data obtained is not due to any artefacts that were generated due to the exposure of GONPs to cells.

#### 2.1.3. The Effects of GONPs on the Inflammatory System Biomarker IL-6 Using RAW 264.7 Cells

GONPs under unstimulated conditions induced a significant (*p* < 0.001) upregulation of IL-6 at 15.6 and 31.25 μg/mL respectively, when compared to the control culture that was not exposed to GONP (Figure 1c). Conversely, IL-6 synthesis was not affected when cultures were exposed to GONP concentrations ≥ 62.5 μg/mL. The results representing LPS stimulated cultures without any nanoparticle exposure were not included in the graph due to the large scale of the data.

#### 2.1.4. The Effects of GONPs on the MIPs Chemokines Using RAW 264.7 Cells

##### The Effects of GONPs on MIP-1α Using RAW 264.7 Cells

GONPs in the range of 15.6–62.5 μg/mL in media only notably (*p* < 0.001) increased the synthesis of MIP-1α, compared to the culture control not exposed to GONP (Figure 1d). However, GONPs concentrations ≥ 125 μg/mL significantly decreased the synthesis of MIP-1α compared to the culture control which was not exposed to GONP. The results for control cultures exposed to media in the presence of a mitogen only (803.85 ± 353.70 ng/mL MIP-1α) are not included on the figure.

##### The Effects of GONPs on MIP-1β Using RAW 264.7 Cells

Cultures exposed to GONP concentrations in the range of 15.6–31.25 significantly (*p* < 0.001) upregulated the amount of MIP-1β secreted by the RAW cells compared to the culture control not exposed to GONP (Figure 1e). GONP concentrations ≥ 62.5 μg/mL, on the other hand significantly reduced MIP-1β synthesis compared to the culture control containing no nanoparticle. Results of media only exposed to LPS in the absence of GONP (1127.19 ± 468.69 ng/mL MIP-1β) are not included in the figure.

##### The Effects of GONPs on MIP-2 Using RAW 264.7 Cells

GONP concentrations ≤ 62.5 μg/mL significantly (*p* < 0.001) increased MIP-2 synthesis compared to the control cultures exposed to media only without any nanoparticle (Figure 1f). MIP-2 synthesis was not affected by GONP concentrations ≥ 125 μg/mL. Media in the presence of LPS without GONP (307.39 ± 171.86 ng/mL MIP-2) is not included on the figure.

#### 2.1.5. Secretory Cytokine and Chemokine Profile of RAW 264.7 Cells UponGONP Treatment

**Figure 2:**
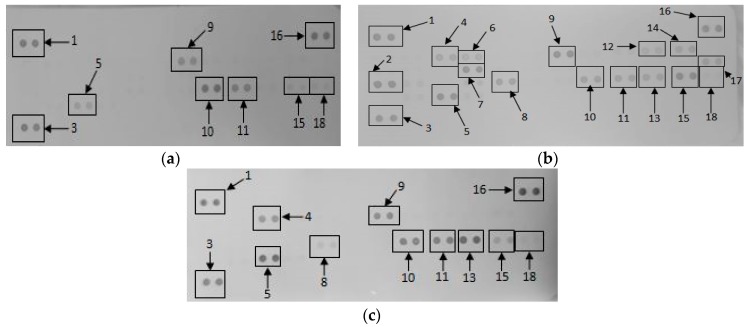
The effect of graphene oxide nanoparticles (GONPs) on RAW 264.7 cells. Cells were incubated with (**a**) media only (negative control); (**b**) media in the presence of LPS and (**c**) 15.6 μg/mL GONPs in the absence of a mitogen. Supernatants were probed using the proteome profiler array as described in methods. Cytokines/chemokines that were detected were allocated numbers: 1, 3 and 16 are reference spots; 2-IP-10; 4-G-CSF; 5-TNF-α; 6-GM-CSF; 7-IL-6; 8-JE; 9-sICAM-1; 10-MIP-1α; 11-MIP-1β; 12-IL-1β; 13-MIP-2; 14-IL-1ra; 15-RANTES; 17-IL-27; 18-SDF-1.

Membranes exposed to media only, media in the presence of a mitogen and 15.6 μg/mL GONPs allowed for the analysis of various cytokines and chemokines expressed by the cells upon the relative exposure (Figure 2). Quantification of the membranes showed that the simulated inflammatory response (media in the presence of LPS) allowed the cells to secrete certain cytokines and chemokines that were not synthesized by the cells when exposed to media only and 15.6 μg/mL GONPs. These proteins include: IFNγ-inducible protein 10 (IP-10); Granulocyte-macrophage colony-stimulating factor (GM-CSF); IL-6; macrophage colony-stimulating factor (M-CSF); interleukin 27 (IL-27); interleukin 1 receptor antagonist (IL-1ra); and IL-β.

**Figure 3:**
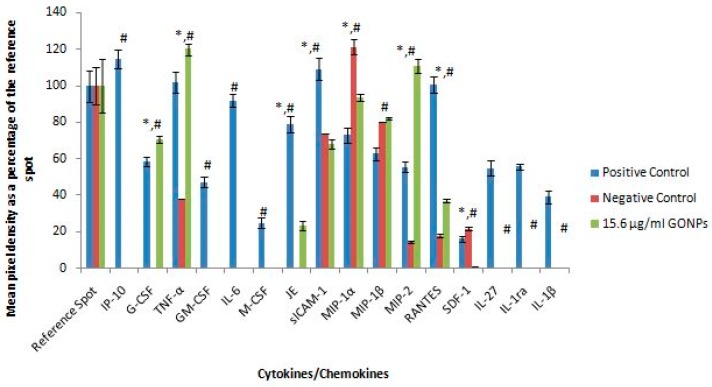
Quantification of cytokines secreted by RAW 264.7 cultures not stimulated with LPS after treatment with medium only or medium containing 15.6 μg/mL GONPs. Membranes subjected to chromogenic exposure. Data is represented as mean ± SD. Bars marked with symbols indicate significant differences (*p* < 0.01). Significance demarcated by: *—significantly different compared to the negative control (*p* < 0.001), ^#^—significantly different compared to the positive control (*p* < 0.001).

However, the exposure of RAW cells to 15.6 μg/mL GONP in the absence of a mitogen affected the synthesis of certain proteins that significantly differed (*p* < 0.001) from both the negative (media only) and positive (media in the presence of LPS) controls. These cytokines and chemokines include: Granulocyte-colony stimulating factor (G-CSF); tumor necrosis factor α (TNF-α); monocyte chemoattractant protein 1 (MCP-1)/JE; intracellular adhesion molecule 1 (ICAM-1); MIP-1α; MIP-2; regulated on activation, normal T cell expressed and secreted (RANTES); and stromal cell-derived factor 1 (SDF-1) (Figure 3).

### 2.2. The Effects of GONPs on Whole Blood Cell Cultures

#### 2.2.1. The Effects of GONPs on Viability of Whole Blood Cell Cultures

**Figure 4:**
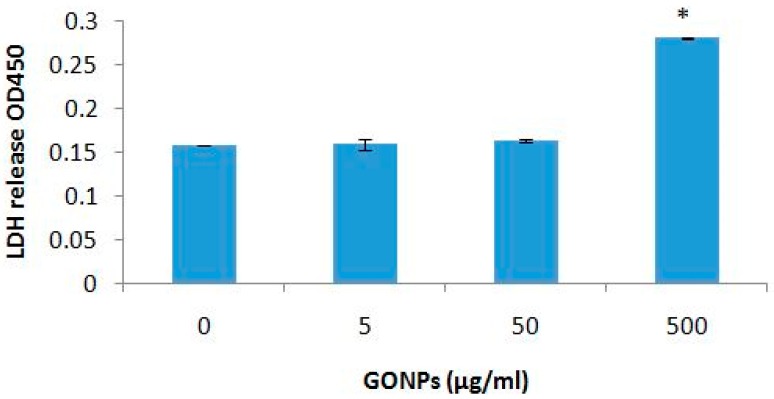
Cell viability of whole blood cell cultures exposed to GONPs. Data represents mean ± SD with *n* = 4. Bars marked with symbol indicate significant difference to control (*p* < 0.01). Significance demarcated by: *—significantly different compared to negative control (*p* < 0.003).

GONP concentrations ≤ 50 μg/mL had no effect on cell viability (Figure 4). However, the highest concentration of GONP evaluated, 500 μg/mL, significantly (*p* < 0.003) reduced viability compared to cultures exposed to media only in the absence of GONP.

#### 2.2.2. The Effects of GONPs on the Inflammatory System Biomarker IL-6 Using Whole Blood Cell Cultures

**Figure 5:**
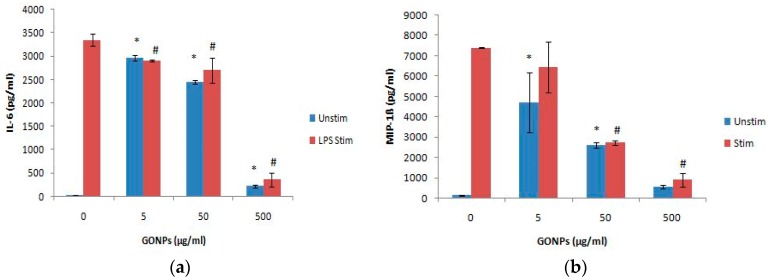
The inflammatory biomarker expression levels of whole blood cell cultures exposed to GO. NPs in the absence or presence of LPS: (**a**) IL-6 expression levels and (**b**) MIP-1β expression levels. Data represents mean ± SD with *n* = 4. Bars marked with symbols indicate significant differences (*p* < 0.01). Significance demarcated by: *—significantly different compared to negative control (*p* < 0.001), ^#^-significantly different compared to positive control (*p* < 0.001).

Cultures exposed to GONPs alone significantly (*p* < 0.001) increased the IL-6 release from the cells at all the concentrations monitored when compared to the culture control exposed to media only (Figure 5a). Conversely, GONPs concentrations in the presence of LPS significantly (*p* < 0.002) reduced the secretion of IL-6 from the cells in a dose dependent manner compared to the control, exposed to media only containing the mitogen.

#### 2.2.3. The Effects of GONPs on the Inflammatory Chemokine, MIP-1β Using Whole Blood Cell Cultures

MIP-1β was significantly (*p* < 0.001) upregulated by GONPs under unstimulated conditions at 5 and 50 μg/mL respectively, when compared to the culture control, not containing any nanoparticle (Figure 5b). However, under a simulated inflammatory response, GONPs exhibited a prominent (*p* < 0.001) reduction in MIP-1β synthesis at concentrations ≥ 50 μg/mL when compared to the culture control, media only containing LPS. 

#### 2.2.4. The Effects of GONPs on the Humoral Immune System Biomarker IL-10 Using Whole Blood Cell Cultures

**Figure 6:**
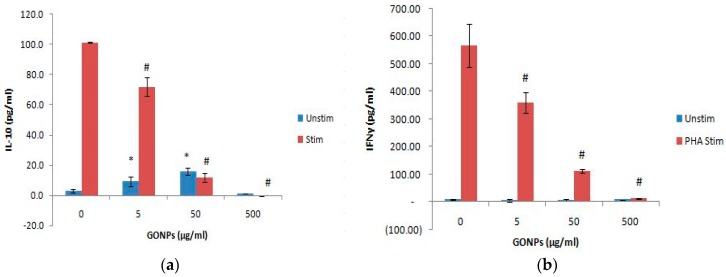
The acquired immune system biomarker expression levels of whole blood cell cultures exposed to GONPs in the absence or presence of PHA: (**a**) IL-10 expression levels and (**b**) IFNγ expression levels. Data represents mean ± SD with *n* = 4. Bars marked with symbols indicate significant differences (*p* < 0.01). Significance demarcated by: *—significantly different compared to negative control (*p* < 0.001), ^#^—significantly different compared to positive control (*p* < 0.001).

GONP concentrations ≤ 50 μg/mL in the absence of PHA significantly (*p* < 0.002) upregulatednIL-10 production compared to the culture control, not containing nanoparticle (Figure 6a). However, the highest concentration (500 μg/mL) of the unstimulated cultures had no effect on the adaptive immune response. On the other hand, GONPs concentrations in the presence of PHA significantly (*p* < 0.001) inhibited IL-10 secretion in a dose dependent manner compared to the culture control, which only contained PHA. 

#### 2.2.5. The Effects of GONPs on the Cell Mediated Immune System Biomarker, IFNγ Using Whole Blood Cell Cultures

GONP concentrations did not affect the cell mediated cytokine, IFNγ under normal conditions (i.e., in the absence of PHA) (Figure 6b). However, GONPs notably (*p* < 0.001) reduced the synthesis of IFNγ in a dose dependent manner in PHA stimulated cultures compared to media only containing PHA (culture control). This dose dependent decrease of IFNγ mimicked the response of the other adaptive immune cytokine, IL-10 (Figure 6a).

#### 2.2.6. Secretory Cytokine and Chemokine Profile of Whole Blood Cell Cultures Upon GONP Treatment

**Figure 7:**
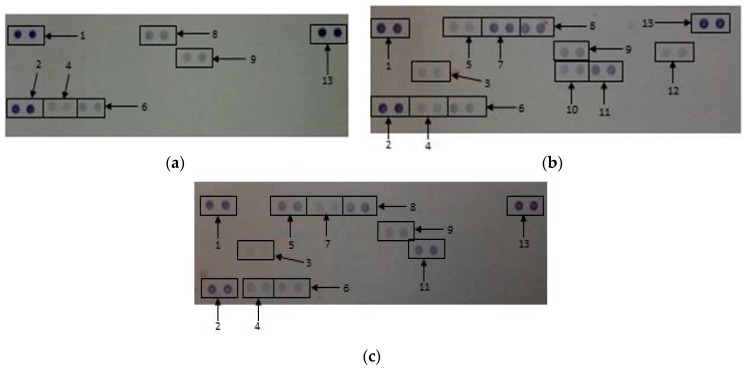
The effects of GONPs on whole blood cells. Cells were incubated with (**a**) media only, (**b**) media and LPS, (**c**) 5 μg/mL GONPs in the absence of LPS. Cytokines/chemokines that were detected were allocated numbers: 1, 2 and 13 are reference spots; 3-IL-1ra; 4-MIF; 5-MCP-1; 6-Serpin E1; 7-MIP-1α/β; 8-RANTES; 9-ICAM-1, 10-IL-6, 11-IL-8 and 12-IL-1β.

The proteome profile of whole blood cell cultures exposed to media only (negative control), media containing the mitogen, LPS (positive control) and 5 μg/mL GONPs in the absence of a stimulus revealed notable changes in the synthesis or inhibition of a number of cytokines and chemokines (Figure 7).

**Figure 8:**
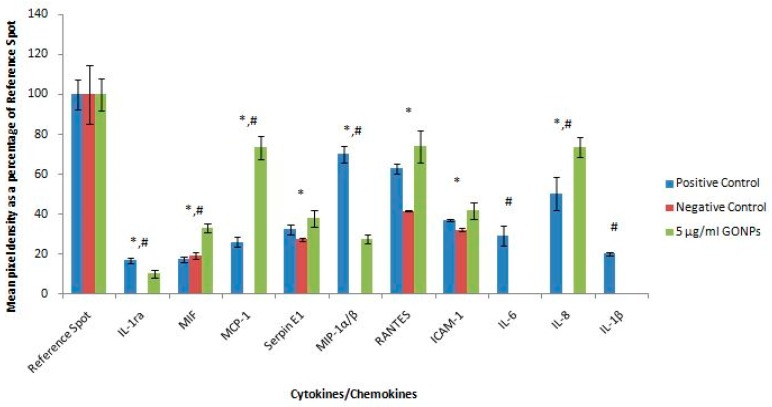
Quantification of cytokines secreted by whole blood cell cultures not stimulated with LPS after treatment with medium only or medium containing 5 μg/mL GONPs. Membranes subjected to chromogenic exposure. Data is represented as mean ± SD. Bars marked with symbols indicate significant differences (*p* < 0.01). Significance demarcated by: *—significantly different compared to the negative control (*p* < 0.001), ^#^—significantly different compared to the positive control (*p* < 0.001).

Figure 8 represents the quantification of the membranes. The whole blood cell cultures exposed to GONPs caused the significant upregulation of macrophage migration inhibitory factor (MIF), MCP-1 and interleukin 8 (IL-8) compared to both controls. The GONPs also notably upregulated serpin E1, RANTES and ICAM-1 compared to the negative control. Cytokines and chemokines that were prominently down regulated compared to the positive control include IL-1ra, MIP-1α/β, IL-6 and IL-1β (Figure 8). Whole blood cells exposed to media only (negative control) did not synthesize the following cytokines: IL-1ra, MCP-1, MIP-1α/β, IL-6, IL-8 and IL-1β.

## 3. Discussion

Graphene has attracted lots of attention due its numerous potential industrial and medical applications. However, very little is known about the effects of these nanoparticles on the immune system. The exposure of RAW cells to GONPs induced cytotoxicity at the highest concentration (500 μg/mL). Similar data was obtained by Li et al. who exposed pristine graphene to RAW 264.7 cells [17]. They proposed that GONPs exerted its cytotoxic effects by depleting the mitochondrial membrane potential and increased the production of ROS. The decrease in cell viability could also be attributed to the higher capacity of macrophages to internalize GO and this could have affected the assembly of actin within the cell [18,19]. A reduction in cell viability was also reported when exposing human THP-1 cells to GONPs, indicating consistent cytotoxic effects against macrophages and monocytes regardless of species [4].

In the current study, inflammatory and proteome profiling data for GONPs, at basal conditions, increased the synthesis of inflammatory cytokines and chemokines such as NO, IL-6, TNF-α, MIPs and RANTES. These findings were corroborated by Orecchioni et al. as it was reported that, GONPs significantly stimulated the secretion of Th_1_/Th_2_ cytokines, such as IL-1a, IL-6, IL-10, TNF-α and GM-CSF, as well as chemokines such as MCP-1, MIP-1α, MIP-1β and RANTES in primary and immortalized murine macrophages [20]. This indicates activation of the macrophages by GONPs and could potentially be attributed to the interaction of GONPs with the toll-like receptors [21,22]. Feito et al. found that RAW 264.7 cells exposed to poly(ethylene glycol-amine) (PEG) functionalized GO upregulated TNF-α under both basal and LPS stimulated conditions [18]. IL-6 was found to be down regulated at 25 and 100 μg/mL GO in a simulated inflammatory response (+LPS). No differences were seen in IL-6 levels under basal conditions. The TNF-α result obtained by Feito et al. is consistent with what was found in the current study using proteome profiling [18]. The inflammatory cytokine, IL-6 was significantly upregulated at 15.6 and 31.25 μg/mL in the absence of a mitogen in the current study. This is contradictory to what was found by Feito et al but could be attributed to the functionalization of GO [18]. Functionalization of nanoparticles with biocompatible polymers increases their stability under physiological conditions. It also minimizes their interactions with other biomolecules and reduce immunological responses [8,18]. The effects of GONPs on RAW cells, under LPS stimulated conditions, were not evaluated in the current study.

Similarly to the RAW cells, GONPs also induced cytotoxicity in whole blood cell cultures at 500 μg/mL, and Orecchioni et al. exposed GONPs to peripheral blood mononuclear cells (PBMCs) and found that it induced its cytotoxic effects by inducing ROS-mediated apoptosis [6,23]. Zhi et al. also found that GO induced apoptosis in T lymphocytes in a dose dependent manner [24].

Exposing whole blood cell cultures to GONPs also induced the upregulation of the inflammatory cytokines IL-6 and MIP-1β under unstimulated/basal conditions. This activation of the inflammatory response could be attributed to the activation of the complement system which results in the production of anaphylatoxins (proinflammatory mediators). This may occur as GO may be sensed by components of the complement system as soon as GO is introduced into a biological system [25]. The activation of the complement system was studied by Wibroe et al. who found that there was an increase in C5a, which is a marker of the activation of the complement system when exposing human serum to GO [26]. Wibroe et al. also found that GO reduced the synthesis of IL-6 when human serum was stimulated by LPS, which is consistent with what was found in this study [26]. Proteome profiling data obtained in the current study confirmed that inflammatory cytokines and chemokines were activated when whole blood cell cultures were exposed to 5 μg/mL GONPs. Notably, IL-1ra, MCP-1, MIP-1α/β and IL-8 were upregulated by the GONPs.

GONPs not only inhibited an inflammatory response in a dose dependent manner but also modulated cytokines regulating the adaptive immune responses. The GONPs stimulated the synthesis of the humoral immune regulating cytokine, IL-10 but did not affect the cell mediated response regulating cytokine, IFNγ, from whole blood cell cultures under unstimulated/basal conditions. This indicates that GONPs would induce the differentiation of Th_0_ cell to Th_2_ cells. The B-lymphocytes are then stimulated to produce antibodies by interleukin 4 and 10 (IL-4 and IL-10) to combat the exposure to GONPs [27]. However, both IL-10 and IFNγ responses by whole blood cell cultures under PHA stimulated conditions were down regulated by GONPs.

This study clearly indicates that GONPs are cytotoxic at high concentrations and stimulates the activation of inflammatory responses under unstimulated/basal conditions. This activation/stimulation of an inflammatory response could cause autoimmunity to individuals exposed to GONPs [28]. Under unstimulated/basal conditions GONPs would also stimulate a humoral immune response in individuals exposed to them. However, under stimulated conditions mimicking an infection, both humoral and cell mediated responses were down regulated, indicating potential immunosuppression by GONPs exposure to individuals with an infection.

The data obtained in the current study is crucial for risk management of people upon GONP exposure, as these nanoparticles could be used in vivo as drug delivery systems, among other applications. GONPs may also be released into the environment and can potentially impact on human health.

## 4. Materials and Methods 

### 4.1. Synthesis and Characterization of Graphene Oxide Nanoparticles (GONPs)

The graphene oxide nanoparticles were synthesized according to a modified Hummers’ method [29], characterized and obtained from the University of Missouri. Graphene sheets were visualized via transmission electron microscopy (TEM, JEOL 1400, Peabody, MA, USA). Further characterization revealed the particles had a maximum absorbance at 227 nm with ultraviolet-visible spectrophotometry (UV-vis) (Lab Tech, UV 8100B, Hopkinton, MA, USA), with a scanning range 200–600 nm. A number of surface functional groups (i.e., hydroxyl and carboxyl groups) were detected by Fourier-transform infrared spectroscopy (FT-IR) in a Nicolet 4700 FT-IR spectrophotometer (Thermo scientific, Waltham, MA, USA) and the spectra collected over a wave number range from 400 to 4000 cm^−1^. The electrophoretic mobility (EPM) of the nanomaterials was measured with a ZetaSizer Nano ZS (Malvern Instruments, Worcestershire, UK) in various electrolyte solutions by laser Doppler velocimetry. EPMs were converted to zeta-potential using the Smoluchowski equation (Equation (1)) [30]:

*U* = εζ/μ, (1)

where *U* is the electrophoretic mobility, ε is the dielectric constant of the solution, μ is its viscosity and ζ is the zeta potential. Disposable folded capillary cells were employed. Zeta potential indicated a surface potential of −49.2 mV at pH 7 and background ionic strength of 10 mM, NaCl (see Appendix A).

### 4.2. Preparation of GONPs

A 10 mg/mL stock of GONPs in distilled water was prepared. The GONPs were sonicated (QSonica, LLC. Misonixsonicators, XL-200 Series, Newtown, CT, USA) in short busts, one ice for a total of approximately 90 min. Aliquots of the stock solution were frozen at −80 °C until use. Before use in experiments, nanoparticles were thawed and further sonicated in short bursts on ice for 5 min.

### 4.3. RAW 264.7 Cells

#### 4.3.1. RAW 264.7 Macrophage Assay

The murine macrophage cell line, RAW 264.7, was obtained from American Type Culture Collection (ATCC TIB-71, Manassas, VA, USA). The RAW 264.7 cells were cultured in Dulbecco’s modified Eagle’s medium (DMEM, Lonza, Cape Town, South Africa) supplemented with 10% heat inactivated fetal bovine serum (FBS, Hyclone, Little Chalfont, UK), glutamax (Sigma-aldrich, St. Louis, MO, USA), antibiotic/antimycotic (Sigma-aldrich) and gentamicin (Sigma-aldrich). The cells were incubated in a humidified atmosphere of 5% CO_2_ at 37 °C and the cells were sub-cultured every 2–3 days.

The RAW 264.7 cells (1 × 10^5^ cells/mL) were cultured in cell culture treated 48 well plates and incubated in a humidified atmosphere of 5% CO_2_ at 37 °C for approximately 48 h until the cells reached 80–90% confluence. After the incubation period, media was removed and replace with media containing 2.5% FBS. The subsequent procedures occurred in serum free media. The cells were pre-exposed for 2 h to various concentrations of GONPs. Thereafter the cells were left unstimulated and a positive control was also present. The positive control were cells only stimulated by lipopolysaccharide (LPS) (1 µg/mL) without the presence of nanoparticles and this would mimic an inflammatory immune response. The final concentration of FBS/well was 0.5%. Cultures were incubated overnight (~18 h) under standard tissue culture conditions. Culture supernatants were collected for nitric oxide (NO), interleukin 6 (IL-6), macrophage inflammatory protein 1α (MIP-1α), MIP-1β, MIP-2 and proteome profiling analysis.

#### 4.3.2. Cytotoxicity Assay

After the removal of the supernatants, cells were washed with Dulbecco’s Phosphate Buffered Saline (DPBS) (Lonza), supplemented with glutamax, antibiotic/antimycotic solution. Cytotoxicity was measured by adding 150 µL of a 1/10 dilution of 2-(4-Iodophenyl)-3-(4-nitrophenyl)-5-(2,4-disulfophenyl)-2*H*-tetrazolium (WST-1, Roche, Basel, Switzerland) reagent in serum free medium to each well. Metabolically active cells convert WST-1 reagent to a formazan that can be measured spectrophotometrically. Formazan formation was determined by reading the plate at 450 nm (Multiskan Ex, Thermo Electron Corporation, Waltham, MA, USA) immediately after WST-1 addition and again after an incubation period of 1 h at 37 °C. The increase in absorbance at 450 nm is proportional to formazan formation. The level of fomazan formed is directly proportional to cell viability.

#### 4.3.3. NO Determination

After the overnight incubation of the RAW 264.7 cells, the amount of nitrite that was produced by the cells was measured in the culture supernatant as an indication of NO production. The NO assay is based on the Griess reaction [31]. The amount of NO production was measured against a doubling dilution range of a 100 μM nitrite standard (Sigma-aldrich). Nitrite standards or culture supernatant collected (100 µL) were mixed with 100 µL of Griess reagent (1:1 of 1% sulfanilamide and 0.1% naphtylethlemidimine-dihydrochloride in 2.5% phosphoric acid) (all reagents obtained from Sigma-aldrich). Thereafter, the plate was incubated at room temperature for 15 min. The absorbance was read at 540 nm by a microplate reader (Multiskan Ex, Thermo Electron Corporation) and the amount of NO produced by the RAW cells quantified.

#### 4.3.4. Mouse IL-6 Double Antibody Sandwich (DAS) Enzyme Linked Immunosorbent Assay (ELISA)

The mouse IL-6 ELISA (e-Bioscience, Ready-Set-Go, Waltham, MA, USA) kits were used to measure IL-6 cytokine levels in the cell culture supernatants. The LPS stimulated control was assayed at (1/40) while the negative control (not treated with LPS) was assayed at (1/5) in assay diluent. Assays were performed in 96 well Nunc maxisorb plates. The kit contained all the reagents for the assay and was performed as per the manufacturer’s instructions (see addendum).

#### 4.3.5. Mouse MIPs (MIP-1α, MIP-1β and MIP-2) DAS ELISAs

Mouse MIP-1α, MIP-1β and MIP-2 ELISAs (R & D Systems, Minneapolis, MN, USA) were performed on the samples and the LPS stimulated culture supernatants. The kits contained all the reagents required for the experiment and experiments were performed as per the manufacturer’s instructions. The samples were all diluted in reagent diluent, 1% human serum albumin (HSA) (*w*/*v*). The MIP-1α samples were assayed at 1/270 and the LPS stimulated supernatant at 1/10,000. For the MIP-1β ELISA, the unstimulated culture supernatants were assayed at 1/20 while the LPS stimulated supernatants were assayed at 1/5000. The MIP-2 ELISA unstimulated supernatants were assayed at 1/100 and the mitogen stimulated supernatant at 1/1000.

#### 4.3.6. Mouse Proteome Profiling Assay

A commercially available antibody array kit (Proteome Profiler, Mouse cytokine Array Panel A, R & D Systems) which was coated with 40 capturing antibodies in duplicate on a nitrocellulose membrane (dot blot) was used. The kit contained all the reagents for the assay and was performed as per the manufacturer’s instructions. This cytokine and chemokine antibody array was used to determine the effects of GONPs exposure on cytokine and chemokine by RAW 264.7 macrophage cells.The assay required 500 μL of cell culture supernatants (unstimulated 0 μg/mL GONPs, LPS stimulated 0 μg/mL GONPs and unstimulated 15.6 μg/mL GONPs). Membranes were subjected to an ultra-sensitive chromogenic 3,3′,5,5′-Tetramethylbenzidine (TMB) membrane substrate (Thermo Scientific, Waltham, MA, USA) to reveal sample-antibody complexes labeled with streptavidin-HRP. Photographs were taken of the blots after the exposure to the substrate.

#### 4.3.7. Quantification of Pixel Density for Cytokine and Chemokine Membranes

Membrane images were quantified using image processing and analysis Java software(version 1.6.0_24, Oracle Corporation, Redwood city, CA, USA), ImageJ (version 1.4.3.67, National Institutes of Health, Bethesda, MD, USA). Levels of cytokines and chemokines were expressed as a percentage of the reference spot. Microsoft Excel was used to calculate the percentages which is expressed as mean ± standard deviation (SD).

### 4.4. Whole Blood Cell Culture

#### 4.4.1. Blood Collection

Blood was collected by a doctor/nurse from a healthy male who was not using any medication. The blood was collected using venipuncture directly into 3.2% sodium citrate vacuum tubes (Greiner bio-one). The blood was processed immediately. The whole blood cell cultures were performed under sterile conditions. Ethical clearance was obtained from the University of the Western Cape (Ethics No. 10/9/43). Informed consent was also obtained from the participant.

#### 4.4.2. Cell Culture

Human whole blood was diluted with Roswell Parks Memorial Institute (RPMI) 1640 media (Sigma-aldrich) to give rise to a 10% (*v*/*v*) mixture. Blood was either left unstimulated or stimulated with LPS (Sigma-aldrich) (0.1 μg/mL) or phytohaemmagglutinin (PHA) (Sigma-aldrich) (1.6 μg/mL). The stimulated conditions would mimic an inflammatory and acquired immune response respectively. Unstimulated or stimulated whole blood cell cultures were incubated overnight at 37 °C with high, intermediate and low concentrations of GONPs in a 24 well tissue culture treated plate (Nunc, Waltham, MA, USA). Along with the nanoparticle exposure, a positive control of 0.01% Tween20 (Merck, Modderfontein, South Africa) was also present. After the incubation period, culture supernatants were screened for cytotoxicity by a lactate dehydrogenase (LDH) assay, inflammatory markers IL-6 and MIP-1β, interleukin 10 (IL-10) and interferon gamma (IFNγ).

#### 4.4.3. LDH Assay

After the overnight incubation, an aliquot of the unstimulated whole blood cell culture supernatants were screened for cytotoxicity and measured the release of LDH from the cells (LDH-cytotoxicity colorimetric kit II, BioVision, Milpitas, CA, USA). The kit contained all the reagents for the assay and was performed as per the manufacturer’s instructions.

#### 4.4.4. Cytokine Analysis Using DAS ELISAs

Commercially available kits (e-Bioscience, Ready-Set-Go) were used to analyze the level of cytokine secretion from the whole blood cell cultures. The kits were used as per the manufacturer’s instructions and contained all the reagents to complete the assay. The unstimulated and LPS stimulated samples were analyzed using a 1/10 dilution for the IL-6 assay. While the unstimulated and PHA stimulated samples were assayed neat for IL-10 and IFNγ analysis. The same protocol was used as previously described for the mouse cytokine ELISA.

#### 4.4.5. Human MIP-1β DAS ELISAs

A human MIP-1β ELISA (R & D Systems) was performed on the unstimulated and LPS stimulated culture supernatants of the whole blood cell cultures. The samples were diluted 1/10 in reagent diluent, 0.1% bovine serum albumin (BSA) (Sigma-aldrich). The same protocol was followed as the mouse MIPs ELISAs.

#### 4.4.6. Human Proteome Profiling

A commercially available antibody array kit (Proteome Profiler, Human Cytokine Array Kit, R & D Systems) which was coated with 36 capturing antibodies in duplicate on a nitrocellulose membrane (dot blot) was used. The kit contained all the reagents for the assay and was performed as per the manufacturer’s instructions. This cytokine and chemokine antibody array was used to determine the effects of GONPs on cytokine and chemokine secretion when exposed to whole blood cell cultures. The assay required 500 μL of cell culture supernatants (unstimulated 0 μg/mL GONPs, LPS stimulated 0 μg/mL GONPs and unstimulated 5 μg/mL GONPs). The subsequent steps were carried out as described for the mouse cytokine and chemokine proteome profiling.

### 4.5. Statistical Analysis

All experiments were performed in triplicate and the data was calculated using Microsoft Excel. Data is presented as mean ± standard deviation (SD). One way analysis of variance (ANOVA) using SigmaPlot 12.0 (Systat Software Inc., San Jose, CA, USA) was used to assess statistical differences with *p* < 0.01 being deemed significant.

## Figures and Tables

**Figure A1:**
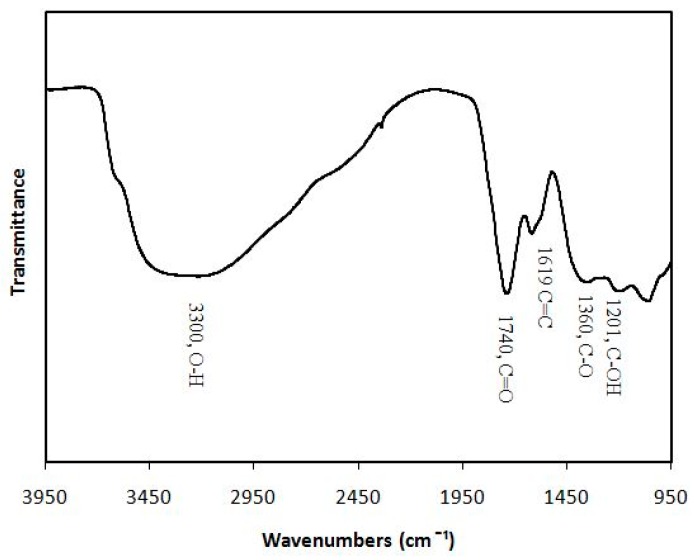
Fourier-transform infrared spectroscopy (FTIR) analysis of GONPs.

**Figure A2:**
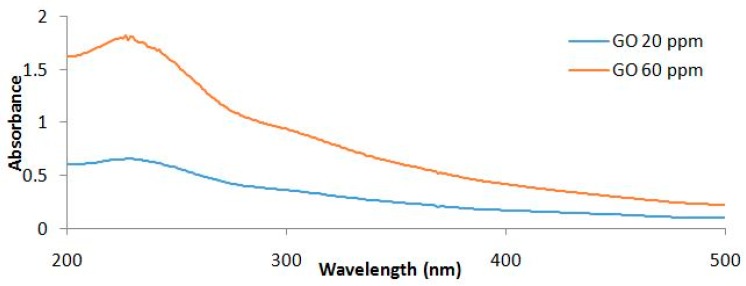
Ultraviolet-visible spectrophotometry (UV-vis) of GONPs, with maximum absorbance at 227 nm.

**Figure A3:**
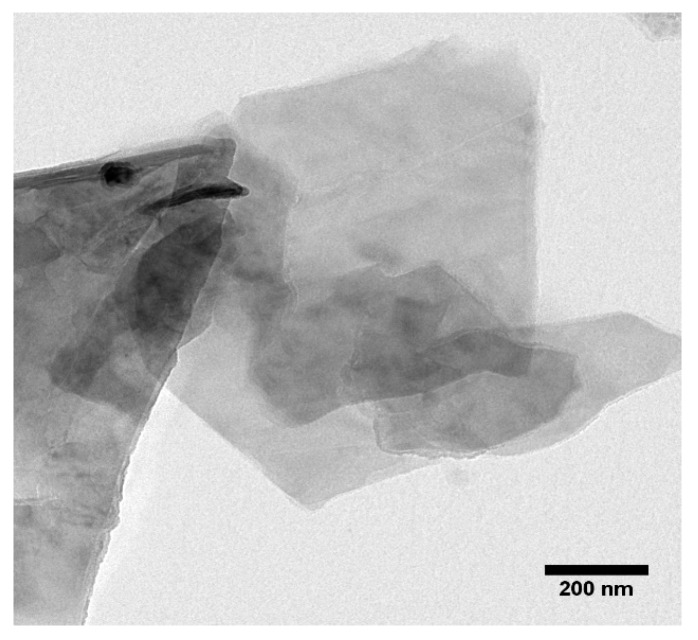
Visualization of graphene sheets via transmission electron microscopy (TEM).

**Figure A4:**
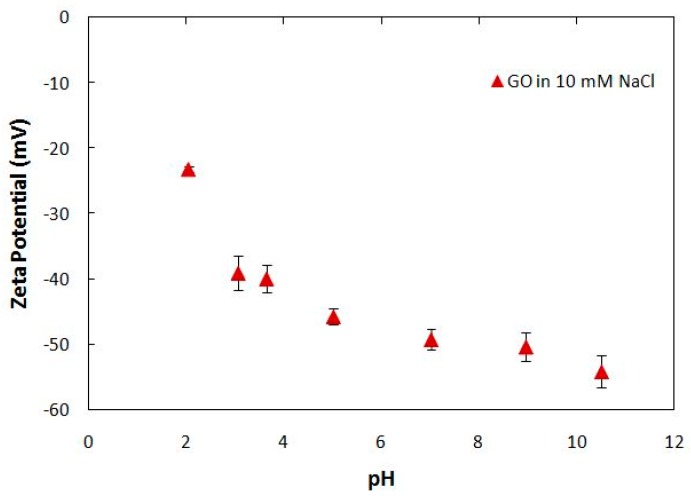
Zeta potential of GONP under varying pH levels, while maintaining a constant ion concentration of 10 mM NaCl.

## Appendix A

The FTIR spectra of GO is presented in Figure A1. Different types of oxygen functionalities were confirmed in the GO spectrum: at 3300 cm^−1^ (O–H stretching vibration), at 1740 cm^−1^ (stretching vibration from C=O), at 1619 cm^−1^ (C=C stretch), at 1360 cm^−1^ (C–O stretch), at 1201 cm^−1^ (C–OH stretching vibration) and finally at 1060 cm^−1^ (C–O stretching vibration). The UV-vis adsorption spectra of the GO sheets showed a characteristic peak at 227 nm (Figure A2) due to the π→π* transition of the C=C bonds. Figure A3 shows a representative TEM image of a GO particle, settled from a suspension at pH = 5.2 and absence of added electrolytes, evidencing its two-dimensional morphology. The dimensions of the sheets observed were about 1–2 µm, although some smaller particles probably from partial breaking of the initial GO sheets were detected. However, in the presence of 10 mM NaCl, the size of the GO sheets decreased from 1555 ± 248.57 nm to 519.97 ± 36.59 nm as a pH increased from 2 to 11 (Table A1). Figure A4 shows that the *ζ*-potential of GO particles was negative over the pH range from 2 to 11. In the presence of 10 mM NaCl, ζ-potential values decreased from −23.24 ± 0.72 to −51.1 ± 1.04 mV as a pH increased from 2 to 11 due to the deprotonation of oxygen functional groups [32]. The *ζ*-potential vs. pH curve displayed two inflection points, located at around pH 4 and pH 9. FTIR spectra (Figure A1) showed that GO has two types of acidic groups: carboxylic (pKa, 4.3) and hydroxylic (pKa, 9.3); increased negative charges on GO sheets resulted from the ionization of these two groups [33].

**Table A1:** Average size of GONP sheets, under varying pH levels, while maintaining a constant ion concentration of 10 mM NaCl.

pH	Average Size (nm)	Standard Deviation
2.05	1555	248.57
3.07	750.47	256.37
3.65	510.43	19.77
5.02	507.47	112.34
7.02	540	10
10.51	519.97	36.59

## References

[1] Chen M., Yin J., Liang Y., Yuan S., Wang F., Song M., Wang H. (2016). Oxidative stress and immunotoxicity induced by graphene oxide in zebrafish. Aquat. Toxicol..

[2] Chen X., Hai X., Wang J. (2016). Graphene/graphene oxide and their derivatives in the separation/isolation and preconcentration of protein species: A review. Anal. Chim. Acta.

[3] Liu B., Salgado S., Maheshwari V., Liu J. (2016). DNA adsorbed on graphene and graphene oxide: Fundamental interactions, desorption and applications. Curr. Opin. Colloid Interface Sci..

[4] Lu C.-J., Jiang X.-F., Junaid M., Ma Y.-B., Jia P.-P., Wang H.-B., Pei D.-S. (2017). Graphene oxide nanosheets induce DNA damage and activate the base excision repair (BER) signaling pathway both in vitro and in vivo. Chemosphere.

[5] Sotirelis N.P., Chrysikopoulos C.V. (2017). Heteroaggregation of graphene oxide nanoparticles and kaolinite colloids. Sci. Total Environ..

[6] Cherian R.S., Sreejith R., Syama S., Sruthi S., Gayathri V., Maekawa T., Sakthikumar D., Mohanan P. (2014). Evaluation of Toxicity of Maura Reduced Graphene Oxide Using in Vitro Systems. J. Nanomed. Nanotechnol..

[7] Zhang W., Yao Y., Li K., Huang Y., Chen Y. (2011). Influence of dissolved oxygen on aggregation kinetics of citrate-coated silver nanoparticles. Environ. Pollut..

[8] Peruzynska M., Cendrowski K., Barylak M., Tkacz M., Piotrowska K., Kurzawski M., Mijowska E., Drozdzik M. (2017). Comparative in vitro study of single and four layer graphene oxide nanoflakes—Cytotoxicity and cellular uptake. Toxicol. In Vitro.

[9] Yan J., Chen L., Huang C.-C., Lung S.-C.C., Yang L., Wang W.-C., Lin P.-H., Suo G., Lin C.-H. (2017). Consecutive evaluation of graphene oxide and reduced graphene oxide nanoplatelets immunotoxicity on monocytes. Colloids Surf. B Biointerfaces.

[10] Hibi M., Nakajima K., Hirano T. (1996). IL-6 cytokine family and signal transduction: A model of the cytokine system. J. Mol. Med..

[11] Niemand C., Nimmesgern A., Haan S., Fischer P., Schaper F., Rossaint R., Heinrich P.C., Muller-Newen G. (2003). Activation of STAT3 by IL-6 and IL-10 in Primary Human Macrophages Is Differentially Modulated by Suppressor of Cytokine Signaling 3. J. Immunol..

[12] Donnelly R.P., Dickensheets H., Finbloom D.S. (1999). The interleukin-10 signal transduction pathway and regulation of gene expression in mononuclear phagocytes. J. Interferon Cytokine Res..

[13] Riley J.K., Takeda K., Akira S., Schreiber R.D. (1999). Interleukin-10 Receptor Signaling through the JAK-STAT Pathway requirement for two distinct receptor-derived signals for anti-inflammatory action. J. Biol. Chem..

[14] Schroder K., Hertzog P.J., Ravasi T., Hume D.A. (2004). Interferon-γ: An overview of signals, mechanisms and functions. J. Leukoc. Biol..

[15] Mühl H., Pfeilschifter J. (2003). Anti-inflammatory properties of pro-inflammatory interferon-γ. Int. Immunopharmacol..

[16] Akhavan O., Ghaderi E. (2010). Toxicity of graphene and graphene oxide nanowalls against bacteria. ACS Nano.

[17] Li Y., Liu Y., Fu Y., Wei T., Le Guyader L., Gao G., Liu R.-S., Chang Y.-Z., Chen C. (2012). The triggering of apoptosis in macrophages by pristine graphene through the MAPK and TGF-beta signaling pathways. Biomaterials.

[18] Feito M., Vila M., Matesanz M., Linares J., Gonçalves G., Marques P., Vallet-Regí M., Rojo J., Portolés M. (2014). In vitro evaluation of graphene oxide nanosheets on immune function. J. Colloid Interface Sci..

[19] Zhang B., Wei P., Zhou Z., Wei T. (2016). Interactions of graphene with mammalian cells: Molecular mechanisms and biomedical insights. Adv. Drug Deliv. Rev..

[20] Orecchioni M., Ménard-Moyon C., Delogu L.G., Bianco A. (2016). Graphene and the immune system: Challenges and potentiality. Adv. Drug Deliv. Rev..

[21] Chen P., Kanehira K., Taniguchi A. (2013). Role of toll-like receptors 3, 4 and 7 in cellular uptake and response to titanium dioxide nanoparticles. Sci. Technol. Adv. Mater..

[22] Mano S.S., Kanehira K., Taniguchi A. (2013). Comparison of cellular uptake and inflammatory response via toll-like receptor 4 to lipopolysaccharide and titanium dioxide nanoparticles. Int. J. Mol. Sci..

[23] Orecchioni M., Jasim D.A., Pescatori M., Manetti R., Fozza C., Sgarrella F., Bedognetti D., Bianco A., Kostarelos K., Delogu L.G. (2016). Molecular and genomic impact of large and small lateral dimension graphene oxide sheets on human immune cells from healthy donors. Adv. Healthc. Mater..

[24] Zhi X., Fang H., Bao C., Shen G., Zhang J., Wang K., Guo S., Wan T., Cui D. (2013). The immunotoxicity of graphene oxides and the effect of PVP-coating. Biomaterials.

[25] Saleem J., Wang L., Chen C. (2017). Immunological effects of graphene family nanomaterials. NanoImpact.

[26] Wibroe P.P., Petersen S.V., Bovet N., Laursen B.W., Moghimi S.M. (2016). Soluble and immobilized graphene oxide activates complement system differently dependent on surface oxidation state. Biomaterials.

[27] Tan T.T., Coussens L.M. (2007). Humoral immunity, inflammation and cancer. Curr. Opin. Immunol..

[28] De Jong W.H., Van Loveren H. (2007). Screening of xenobiotics for direct immunotoxicity in an animal study. Methods.

[29] Humers W., Offeman R. (1958). Preparation of graphitic oxide. J. Am. Chem. Soc..

[30] Elimelech M., Gregory J., Jia X., Williams R. (1995). Particle Deposition and Aggregation Measurement, Modeling and Simulation.

[31] Granger D.L., Taintor R.R., Boockvar K.S., Hibbs J.B. (1996). Measurement of nitrate and nitrite in biological samples using nitrate reductase and Griess reaction. Methods Enzymol..

[32] Chowdhury I., Duch M.C., Mansukhani N.D., Hersam M.C., Bouchard D. (2013). Colloidal properties and stability of graphene oxide nanomaterials in the aquatic environment. Environ. Sci. Technol..

[33] Zhao J., Liu F., Wang Z., Cao X., Xing B. (2015). Heteroaggregation of graphene oxide with minerals in aqueous phase. Environ. Sci. Technol..

